# A shifting baseline theory of debates over potential lynx and wolf reintroductions to Scotland

**DOI:** 10.1007/s13280-025-02186-w

**Published:** 2025-04-22

**Authors:** Toryn Whitehead, Darragh Hare

**Affiliations:** 1 Department of Geography, King’s College London, 40 Aldwych, London, WC2B 4BG UK; 2 Wildlife Conservation Research Unit, Department of Biology, The Recanati-Kaplan Centre, University of Oxford, Oxford, UK; 3 Leverhulme Centre for Nature Recovery, School of Geography and the Environment, University of Oxford, Oxford, UK; 4 Department of Natural Resources and the Environment, Cornell University, Ithaca, NY USA

**Keywords:** Human–wildlife coexistence, Lynx, Reintroduction, Rewilding, Shifting baseline syndrome, Wolf

## Abstract

In Scotland, efforts to reintroduce extirpated species have been marred by guerrilla rewilding and social conflicts. We ask whether these conflicts could at least in part be the product of shifting baseline syndrome. The multi-generational absence of many large charismatic species has resulted in an ‘extinction of experience’ about how to coexist with them embedded within the structures, institutions, and cultural products of Scottish landscapes. We draw on academic literature, popular media, and policy documents to consider debates over the potential reintroduction of the Eurasian lynx (*Lynx lynx*) and wolf (*Canis lupus*) to Scotland from a shifting baseline perspective. The paucity of (scientific and historical) knowledge about the social and ecological impacts of locally extinct species and the loss of coexistence experience has created more fertile ground for myths and wishful thinking to proliferate uninhibited, resulting in the romantic and cynical cultural transformation of the lynx and wolf in Scottish society. We argue that empathetic, patient, and transparent dialogue can help to co-produce shared visions of rural landscapes, with or without large carnivores, which retain ecological ambition and support multiple land-use systems, while ensuring that any transitions are socially just and economically feasible.

## Introduction

“The truth is we know little about the wolf. What we know a good deal more about is what we imagine the wolf to be” (Lopez 1978, p. 3).

Historical land-use changes in Scotland, such as the highland clearances and the rise of Victorian sporting estates, have produced highly concentrated patterns of private land ownership (Hunter 2015; Smout 2019). Simultaneously, these land-use changes have favoured extensive livestock grazing systems, grouse production, and the proliferation of native red deer (*Cervus elaphus*) and roe deer (*Capreolus capreolus*) populations, which combined number more than one million (Pepper et al. 2020). The cumulative legacy of these historical land-use changes is that Scotland has a Biodiversity Intactness Index of 45%, lower than any G7 country (range 62–91%) and other northern European countries such as Sweden (94%) and Norway (95%) (Walton et al. 2023). Yet, a history of conflicts over how rural Scottish landscapes should look, what purposes they should serve, and who should control them has resulted in widespread distrust among multiple rural interests, largely falling along social identity lines—making collective action to counteract biodiversity loss very difficult (Ainsworth et al. 2020; Hare et al. 2021; Holmes et al. 2024).

Rewilding has emerged internationally as one proposed solution which advocates large-scale restoration of rural landscapes to develop self-regulating resilient ecosystems (Carver et al. 2021). The reintroduction of extirpated (locally extinct) species is a central pillar of this strategy. Scotland has a long history of reintroductions, from the capercaillie (*Tetrao urogallus*) in 1837 to more recent reintroductions of red kites (*Milvus milvus*), white-tailed eagles (*Haliaeetus albicilla*), Eurasian beavers (*Castor fiber*), and wild boar (or feral pigs) (*Sus scrofa*). The growth of raptor populations has been characterised by illegal killings and hostility between actors (Evans et al. 2009; Smart et al. 2010; Hodgson et al. 2018; Marino et al. 2023). Beavers and wild boar have also sparked social conflicts, with their illegal reintroduction precipitating widespread legal and illegal persecution (Holmes et al. 2024). That being said, the illegal reintroduction of beavers in the River Tay catchment burst the political dam and forced the Scottish government to grapple with their presence (Thomas 2022), making way for further beaver reintroductions across the UK. While this could be considered a rewilding success, illegal wildlife reintroductions have eroded trust and strained relationships between land managers and conservationists in Scotland, as well as undermining evidence-based conservation and politicising conservation translocations and the animals themselves who are translocated (Whitehead 2025; Sutherland et al. 2025).

The reintroduction of LCs, particularly Eurasian lynx *(Lynx lynx*), is a major objective for many rewilding advocates in Scotland*.* At present, *The Missing Lynx Project* is exploring the feasibility of a lynx reintroduction in Northumberland, England, into a habitat patch which also includes bordering areas of southern Scotland. The Lynx to Scotland project, comprising three rewilding organisations, proposes a phased release programme with annual check points, over at least a five-year period, in the Scottish Highlands. The legal reintroduction of lynx by either of these projects was preceded by the illegal release and subsequent capture of four lynx in the Cairngorms National Park in the Scottish Highlands in January 2025, possibly by guerrilla rewilders. While there are no active proposals to introduce wolves (*Canis lupus*) in Scotland, the idea was first mooted in the 1990s and received further attention with discussions of reintroducing wolves to an enclosure in Alladale Wilderness Reserve around 2010 (Taylor 2011). There also appears to be a growing interest in wolves from certain quarters of the rewilding community, evident from the publication of Hunt for the Shadow Wolf (2024) and the first wolf conference in the UK which convened wolf enthusiasts and rewilding experts in 2024. Beyond Scotland, lynx and wolves have made a remarkable comeback in mainland Europe, expanding their populations and ranges to areas where they have been absent for multiple generations (Linnell et al. 2009; Blanco and Sundseth 2023). Therefore, while our focus remains strictly on Scotland, we draw on literature from across Europe to help build our argument and believe that our framework could have merit in European and other contexts.

**Fig. 1:**
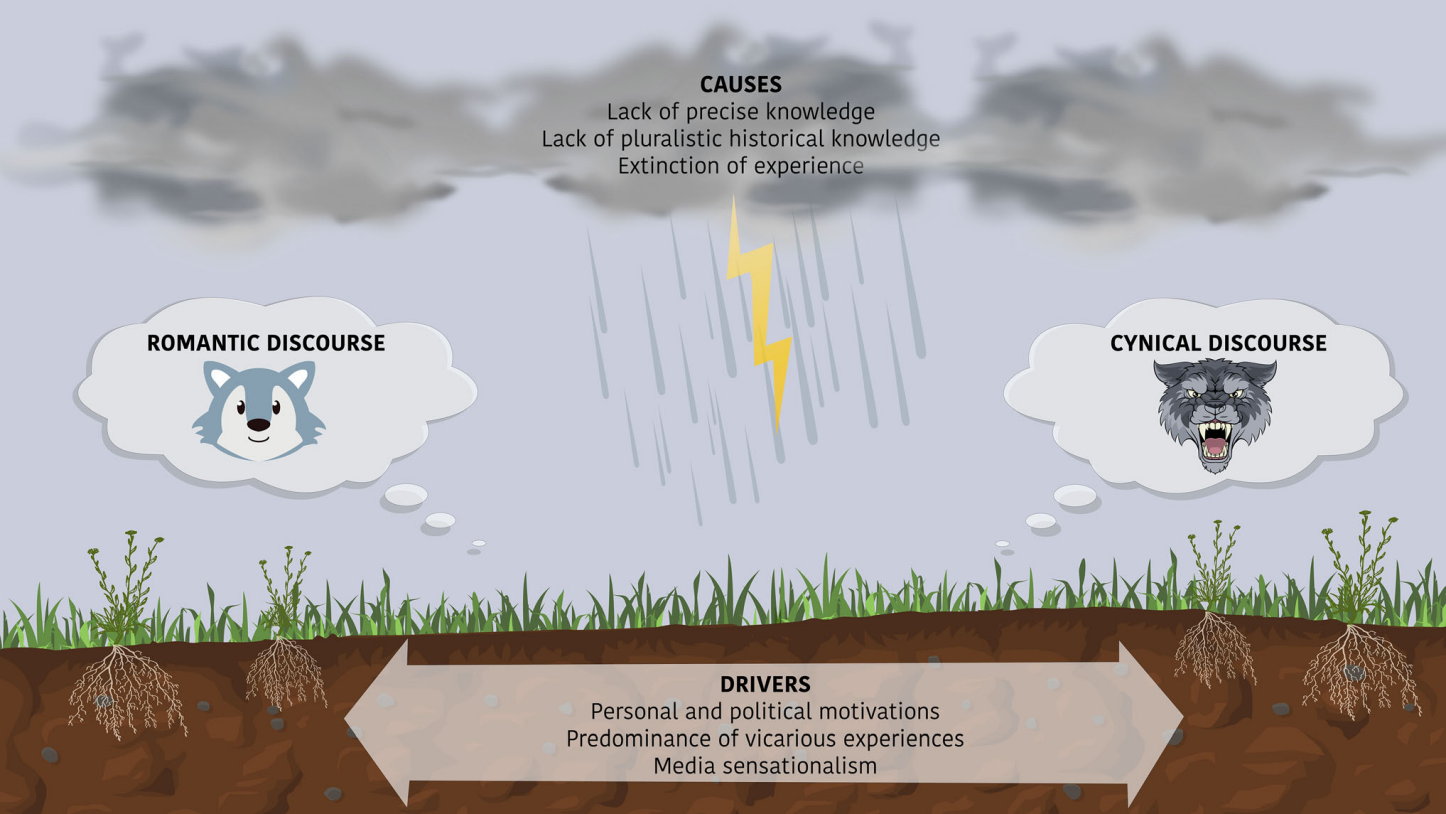
A theoretical framework for the cultural transformation of extirpated species, in this instance, wolves. The ‘causes’ create more fertile ground for myths and wishful thinking to germinate and propagate. The ‘drivers’ are simultaneously the sources of misinformation and help to create a more favourable climate for propagation. The combination of the ‘causes’ and ‘drivers’ has produced a perfect storm from which the romantic and cynical discourse have emerged

Here we ask whether divisions over extirpated species reintroductions in Scotland, linked to an emerging and polarising rewilding agenda, could be at least in part the product of shifting baseline syndrome (SBS). To answer this question, we draw on academic literature about SBS and human–wildlife coexistence, popular media, and policy documents to synthesise a framework which maps out the cultural transformation of LCs in Scotland (Fig. 1). Building on Jarić et al. (2022), cultural transformation here can be understood as the metamorphosis of a biologically extirpated or extinct animal to an ‘imagined’ animal which is estranged from reality and embodies certain human values. Inspired by Soga and Gaston’s (2018) framework which outlines three primary causes of SBS, our framework identifies three causes of this cultural transformation process: (1) the lack of precise knowledge about the impacts of LCs; (2) the lack of pluralistic historical knowledge; and (3) the extinction of experience. Importantly though, these causes only create more favourable conditions for a cultural transformation to occur and do not necessarily drive the cultural transformation process itself. Therefore, we also identify three possible drivers of the cultural transformation of LCs in Scotland: (i) personal and political motivations; (ii) the predominance of vicarious experiences; and (iii) media sensationalism. We hypothesise that the causes and drivers together have culturally transformed LCs, resulting in the emergence of the romantic and cynical discourses (see Box 1 and Box 2). That being said, we recognise the plurality of perspectives that exist relating to debates over LCs and do not want to deny or occlude that complexity by identifying two divergent discourses. However, we believe thinking in terms of two general discourses (romantic and cynical) can offer a useful heuristic to characterise dynamic divisions over the possibility of LC reintroductions to Scotland.

Box 1 A brief synopsis of the romantic discourse The** romantic discourse** is driven by a sense of urgency to act on the climate and biodiversity crises, and the return of LCs is of paramount importance to this. However, the restorative powers of LCs and their ability to control high deer populations have frequently been overstated, and human–LC coexistence in Europe has often been idyllically portrayed. While this discourse has engaged with some of the social, economic, and cultural aspects of coexistence, advocates have failed to work transparently and collaboratively to demonstrate how these challenges could be overcome in a Scottish context.

Box 2 A brief synopsis of the cynical discourse The** cynical discourse** portrays the reintroduction of LCs as an existential threat to livestock farming, recreational hunting, and by extension, rural culture. While many of these claims may be unfounded, there is often a kernel of truth within them relating to the substantial challenges a LC reintroduction, particularly wolves, would pose for traditional rural livelihoods. For proponents of this discourse, LCs are intrinsically linked to rewilding, the blueprint for which, in their view, has been illegal releases of beavers, wild boar, and lynx.

## Cultural transformations of lost species

SBS refers to the gradual decline in accepted norms and expectations for the condition of the natural environment across an individual’s lifetime or across generations (Soga and Gaston 2018). SBS encompasses (i) generational amnesia, where younger and older generations have different perceptions of ecological change (Kahn and Kellert 2002), and (ii) personal amnesia, where an individual’s perception of change does not match the actual changes which have occurred (Papworth et al. 2009). The word amnesia is significant because it draws attention to loss of knowledge being unconscious (Fernández-Llamazares et al. 2015). Thomas (2020) argues that using contemporary baselines for social–ecological conditions to inform land management can overlook how current conditions are (potentially unstable) outcomes of historical processes, inadvertently creating new threats to social-ecological systems.

For example, historical records show that the Dogger Bank Marine Protected Area (MPA) in the North Sea has undergone vast ecological shifts since the early nineteenth century (Plumeridge and Roberts 2017). Despite historical impoverishment, in 2012 the UK Joint Nature Conservation Committee (JNCC) devised restoration objectives for the MPA using data obtained in the previous ten years. By using a contemporary baseline, JNCC ignored two centuries of biodiversity loss and failed to consider the restoration of fish communities—resulting in conservation objectives which normalised a degraded ecosystem (Plumeridge and Roberts 2017).

In the British Isles, the impact of people on the environment can be traced back at least 40 000 years, to a time of straight-tusked elephants (*Palaeoloxodon antiquus*), two species of rhinoceros (*Stephanorhinus kirchbergensis* and *Stephanorhinus hemitoechus*) and giant elk (*Megaloceros giganteus*). While these species and many others, large and small, have become biologically extinct since the Late Pleistocene, most have also become societally extinct due to “the loss of collective memory, attention, knowledge, representations, and cultural products associated with species from cultures and/or societies (Jarić et al. 2022, p. 411). Other species, despite their biological extinction, remain societally extant and, in many instances, are culturally transformed as well.

For example, woolly mammoths (*Mammuthus primigenius*) became extinct nearly 4000 years ago, yet they remain societally salient through cultural products such as books (e.g. *Mammoth Academy* by Neal Layton) and films (e.g. The *Ice Age* franchise). These cultural products often portray woolly mammoths as cuddly and friendly—characteristics that almost certainly would not mimic the *real* animal. The human characteristics and values we superimpose on to species, intentionally or not, often become attached to the species and engrained in society’s collective memory. While woolly mammoths’ portrayal has been positive, it is an example of superficial anthropomorphism—the inappropriate or inaccurate attribution of human characteristics to nonhuman entities (Ferraro et al. 2023). In this instance, the superficial anthropomorphism of woolly mammoths may be harmless, but, as with lynx and wolves, romantic and cynical constructions of nonhuman characteristics and behaviours can be dangerous.

## Causes: Creating fertile ground

### Lack of precise knowledge about the impact of large carnivores

LCs remain extant across Europe, and their populations and ranges have expanded due to legal protections. However, the precise impacts of LCs on extensive livestock grazing systems, wild ungulate populations, and the natural environment remain uncertain. While low transparency and inconsistent record keeping mean the extent of livestock predation is difficult to quantify, it appears the financial impact of predation would be minimal relative to other causes of livestock mortality (Blossey and Hare 2022). However, the extent of livestock predation will depend on the existence and effective implementation of preventative measures. Norway has a relatively small populations of LCs and free-ranging livestock with little supervision. The number of livestock killed each year per wolf is 34 and per lynx is 16 (Blanco and Sundseth 2023). Sweden has a larger population of LCs and more prevention measures. The number of livestock killed each year per wolf is 0.85 and per lynx is 0.1 (Blanco and Sundseth 2023). Although there are critical differences in land ownership, agricultural systems, and governance structures of Scandinavia and Scotland, these comparisons underscore the importance of livestock protection measures. While the return of LCs can sometimes only require inexpensive materials and minor husbandry changes (Blanco and Sundseth 2023), in Scotland, where the national flock is estimated at around 6.47 million head (Scottish Government 2024), it would likely require significant and costly changes to husbandry practices as well as an increase in labour intensity. Without expertise to support transitions and long-term funding to support changes to husbandry practices and effective livestock protection, it is not surprising that livestock producers are unenthusiastic about the possible return of LCs (Bavin et al. 2023; Tan et al. 2023).

There is some evidence that lynx could help maintain already low populations of roe deer, with kill rates and concentration of kills increasing when lynx encounter naïve and abundant prey (Andrén and Liberg 2015; Duľa and Krofel 2020). But reintroducing lynx alone would be unlikely to significantly reduce roe deer numbers or impacts in Scotland (Kirkland et al. 2021). Similarly, wolves’ impact on deer populations depends on the presence of people and deer density (Kuijper et al. 2016), with wolves potentially unable to control densities exceeding 7–8/km^2^ (Pimlott 1967). The reintroduction of wolves to Yellowstone National Park (YNP) in 1995 led to increased predation of ungulates and created a landscape of fear which influenced ungulate behaviour and reduced population densities (Laundré et al. 2001). However, while often heralded as an exemplar of wolf-induced trophic cascades, problems associated with selective evidence collection have cast doubt over the extent of their role (Brice 2022). Furthermore, evidence from across Europe suggests that human hunting pressure is the main driver of red deer density and that a reduction in deer density only occurred when lynx, wolves, and the Eurasian brown bear (*Ursus arctos arctos*) co-occurred within the same site (van Beeck Calkoen et al. 2023). Therefore, LCs are unlikely to be a panacea for Scotland’s deer crisis. This uncertainty is itself not necessarily a barrier to any future LC reintroduction—rewilding interventions must embrace uncertainty and emerging ecological theories emphasise change [e.g. alternative stable states (Hobbs et al. 2024)] (Hawkins et al. 2024). However, uncertainty over the precise impacts of any LC reintroduction means that romantic and cynical notions of LCs are more difficult to quell, potentially allowing them to expand and drive the cultural transformation of LCs.

### Lack of pluralistic historical knowledge

A lack of historical environmental data is a fundamental driver of SBS (Soga and Gaston 2018) and historical records about wolves prove revealing. In the mediaeval period, literacy was heavily linked to power and wealth, so shepherds, farmers, and hunters (who lived in close proximity to wolves and were most likely to have direct interactions with them) were usually illiterate. Therefore, most historical records from this time can be attributed to landowners (Pluskowski 2006). Landowners’ accounts would have been shaped by their interests in hunting, forestry, and agriculture, creating a perceived economic incentive to exterminate wolves. At the same time, Christianity had already established itself as the principal religion in Great Britain. During this time oral storytelling began to impose “a veneer of Christianity on old, non-Christian stories” which vilified wolves (Marshall 2022, p. 128). This was most likely a result of wolves’ close association with Satan, where, for example, wolves were used as a metaphor for Satan in the New Testament in opposition to Christ as a lamb and shepherd. Consequently, selective recreations of the Middle Ages which portray wolves as “bloodthirsty, man-eating creatures of the wilds beyond civilisation” are popular in the Western imagination (Marshall 2022, p. 212).

These selective recreations may not represent experiences of farmers, shepherds and hunters who shared the landscape with wolves. Pluskowski (2006, p. 25) notes that the few records that have survived “indicate a more informed understanding of animal behaviour based on working relationships with both wild and domestic fauna”. In Mesolithic Ireland, people revered wolves as skilled hunters and behavioural role models in conflict, as well as competitors for prey species, a source of food and fur, and important to nature−centred belief systems (Overton and Taylor 2018). Even with the arrival of livestock farming in the Neolithic period, evidence suggests the emphasis was placed on reducing negative impacts of wolf predation (rather than extermination) (Sands 2022). Pluralistic attitudes towards wolves still persisted with the advent of Christianity, as some Irish saints conveyed “a strong ethic of stewardship, care, and reciprocity towards nature and wild animals, including wolves” (Sands 2022, p. 261). Yet, despite this long period of sustained coexistence, accounts from pre−colonial Ireland, characterised by a belief system with more pluralistic ways of relating to nature and wolves, are scarce (Sands 2022). The scarcity of accounts from other belief systems and from individuals who lived in closer proximity to wolves may have contributed to the villainous social construction of wolves in Scottish collective memory.

Although there are several historical records about lynx, such as Richard Pococke’s *Tour of Scotland* (1760) which provides a possible record of lynx by referring to a “wild cat three times as big as the common cat” (Raye 2021), they have not remained societally salient like wolves. This is evident from the lack of folklore and pop culture about lynx. One reason for this could be their elusive nature which has meant that even where they occur, people know very little about them (Lescureux et al. 2011). Another reason could be that where there are wolves and bears, people pay little attention to lynx because of their relatively small impact on livestock. Nevertheless, the lack of historical documents referring to lynx suggests that contemporary (positive and negative) social constructions of lynx are not rooted in historical Scottish experiences.

### The extinction of experience

In Spain, three discourses have emerged relating to contemporary human–wolf coexistence with distinct visions for the future of rural landscapes (Pettersson et al. 2023). The ‘protectionist’ discourse argues that human activities should be significantly reduced to maximise nature’s autonomy and enable wolf populations to flourish freely. The ‘traditional’ discourse argues that wolf populations should be conditioned by local resource systems, prioritising social and cultural rural landscapes instead. The ‘pragmatic’ discourse argues that the costs and benefits of living with wolves should be fairly distributed in order to foster a favourable state of coexistence, balancing conservation goals and local priorities. Pettersson et al. (2023) observed that in areas where wolves had been extirpated the ‘protectionist’ and ‘traditionalist’ discourses were predominant, whereas in areas where wolves had never been extirpated, the pragmatic discourse was common with residents (including shepherds). Residents in these areas were used to living with wolves and so had been exposed to “the complexities of coexistence” (Pettersson et al. 2023, p. 1996). Although there are substantial social, cultural, and economic differences between Spain and Scotland, one factor driving the cultural transformation of LCs in Scotland might be the relative shrinking of the pragmatic discourse as a result of the extinction of experience. But what precisely has gone extinct?

Environmental knowledge and collective memory of species is derived from direct, lived experiences and indirect, vicarious experiences (Soga and Gaston 2018). Lived experiences refer to direct human–nature interactions based on sensory contact, and as such are more closely associated with the biological characteristics of the species (Jarić et al. 2022). Vicarious experiences refer to virtual human–nature interactions where there is no direct sensory contact with the species (Jarić et al. 2022). Folklore, films, social media, the internet, paintings, books, and songs are all examples of vicarious experiences. Since LCs have been absent from Scotland for at least three hundred years, there has been an extinction of direct interactions (with the exception of zoos and safari parks). This is significant because direct interactions are important for forging emotional connections with species and for accumulating traditional and local ecological knowledge, premised upon a long residence in a place (Fernández-Llamazares et al. 2015; Pettersson et al. 2025).

#### The loss of coexistence experience

In North Macedonia, Lescureux et al. (2011) found that participants’ perceptions of lynx tended to be slightly negative—with 68% of livestock breeders perceiving lynx as harmful to livestock. However, only two direct cases of attacks were reported by informants. The speculation that lynx were harmful to livestock might partly have been a consequence of the absence of experience-based and scientific knowledge which created favourable conditions for rumours to develop and expand, for example, that lynx are bloodthirsty and suck the blood of their prey. However, the knowledge acquired from simply sharing the landscape with lynx, as well as the relatively few direct interactions people did have with lynx, appear to have prevented these rumours from becoming prevalent and has produced a broad consensus that lynx pose a low threat to human safety; and that despite a perceived threat to livestock, informants preferred protection over extermination or a reduction in the lynx population. Therefore, although lynx may be elusive, the absence of direct and indirect human–lynx interactions may have provided a greater opportunity for myths and wishful thinking to emerge and spread in different segments of Scottish society.

The tolerance of livestock grazing systems to predation may have also shifted as a consequence of the extinction of experience. Within Italy in areas where wolves have always existed, farmers are more tolerant to livestock predation than farmers in areas where wolves were extirpated but are now recolonising; where “even one sheep loss is intolerable” (Boitani, pers. comm., 2024). One reason for this shift in tolerance could be the loss of intangible benefits, described as positive emotions and non-monetary benefits resulting from coexistence (Marino et al. 2021). Intangible benefits are linked to direct interactions, such as unexpected encounters, and indirect interactions, such as pride of sharing the landscape with LCs. Intangible benefits have a positive relationship with tolerance towards wolves and so their loss could be consequential (Marino et al. 2021; Durá-Alemañ et al. 2024). In addition, wolves’ sustained absence has meant that farmers in these areas no longer have experience of dealing with livestock predation (by wolves) or implementing preventative measures. This extinction of experience is also embedded within livestock grazing systems financially as farmers no longer have to account for losses from wolves or pay for preventative measures. In Scotland, as in those areas of Italy where wolves were extirpated, the extinction of experience may perhaps have contributed to a lower tolerance of, and capacity to coexist with, LCs. Farmers and crofters would have to shoulder additional financial costs and learn—practically and culturally—to coexist with LCs for the first time.

Any visions for the future of Scottish rural landscapes which include LCs must not only consider their reintroduction, but also the features of social and cultural landscapes that are critical for fostering favourable states of coexistence. This aligns with broader literature around the requirement for rewilding to integrate social and cultural changes to support coexistence (Carver et al. 2021; Hawkins et al. 2024) and is exemplified by brown bears in the Pyrenees. Their reintroduction in 1996 was undertaken in a contentious socio-political climate which gave no voice to farmers in the decision-making process (Rubio-Ramon and Syse 2022). In response to increasing populations and predation, the Catalan Government launched a sheep regrouping policy which included protective measures to counteract rising sheep casualties (Ferrer and Pons-Raga 2022). One measure was to provide livestock farmers with a shepherd and herding dog over the summer season, a practice that had virtually disappeared from the area. However, the function of shepherds became subverted, since their primary goal was no longer solely to protect livestock from bears, but also to protect bears from humans’ harmful actions (Rubio-Ramon and Syse 2022). This led to the term ‘wildlife shepherd’, since they simultaneously preserved the cultural and economic value of livestock farming while conserving bears’ ecological and political value (Rubio-Ramon and Syse 2022). As such, wildlife shepherds helped reconcile what farmers deemed an outside conservationist agenda with a ruralist discourse celebrating agropastoral traditions (Rubio-Ramon and Syse 2022). This process of cultural revitalisation in the Pyrenees, rooted in rural communities and local identities, was achievable because shepherds still persisted within society’s collective memory. Furthermore, shepherds’ persistence was embedded in the landscape through structures such as shepherd huts, institutions such as local councils and farming organisations, and cultural products such as sheep cheeses (Rubio-Ramon and Syse 2022; Ferrer and Pons-Raga 2022).

This case study underscores the importance of restoring social and cultural features. However, for this process to be socially just and successful in Scotland, restoration of cultural practices and introduction of new practices will require meaningful collaboration with farmers and other rural interests. The lack of participation by local farmers in the Pyrenees bear program and the sheep regrouping policy led to discontent and strong feelings of imposition (Ferrer and Pons-Raga 2022). Furthermore, despite framing the regrouping policy as a *return* of shepherds, the shepherding model implemented through this policy was very different from preceding practices (Ferrer and Pons-Raga 2022). Framing the policy as a cultural *restoration* was not accurate and irked local farmers, even if it made the project more politically and socially palatable (Ferrer and Pons-Raga 2022). While this may not be a problem in Scotland since memories of LC management practices no longer exist, it still raises important questions about the intrinsic link between the past—*re*wilding and *re*introduction—and contemporary efforts to bring LCs to Scotland.

SBS implies the existence of a singular, universally desirable baseline, which Soga and Gaston (2018) rightly point out does not exist. Similarly, we want to be clear that a universally desirable baseline for human–LC coexistence characterised by relative peace and harmony does not exist. Coexistence is a complex, dynamic social–ecological process (Glikman et al. 2021). For example, while wolves have always remained extant in Estonia, the parameters of coexistence from the Soviet period to today are ever-changing. In 2004, overhunting and poaching reduced the population to six reproductive packs (Veeroja et al. 2024). In 2023, there were 39 reproductive packs and reports of record-high livestock predation (Veeroja et al. 2024). Furthermore, while multi-generational experiences of coexistence may contribute to more neutral and positive attitudes towards LCs in certain cases (Torres et al. 2020), this may not always be the case. In fact, Barmoen et al. (2024) found that people living in areas where wolves had never been extinct have more negative attitudes than people living in areas where there are no wolves, or where wolves have recolonised. Importantly though, people living in areas where wolves have always existed accept that they belong in the landscape and despite more negative attitudes, appear to have a higher tolerance to livestock predation events (Pettersson et al. 2022; Durá-Alemañ et al. 2024). In sum, the existence of a universally desirable baseline for coexistence is a romantic notion, and weaponising SBS to advocate for the reintroduction of LCs based on unfounded, nostalgic imaginations of past landscapes and human–LC relationships would be irresponsible.

## Competing visions of large carnivores and rural landscapes

We contend that the combination of these factors—the lack of precise knowledge, the lack of pluralistic historical knowledge, and the extinction of experience—may have helped to cultivate more fertile ground for myths and wishful thinking about LCs to propagate uninhibited. We hypothesise that the lack of inhibition could be significant because without knowledge derived from multi-generational experiences of coexistence or environmental education, misinformation is more likely to become widespread and embedded within society’s collective memory as a common truth. We posit that three factors have driven LC’s cultural transformation.

First, the predominance of vicarious experiences has meant that people’s knowledge and attitudes towards LCs are informed by sources which are more prone to exaggeration and misinformation. For example, C.S. Lewis’s book *The Lion, The Witch and The Wardrobe* (1950), which was recounted in the *Chronicles of Narnia* TV series (2005), falsely portrays beavers as omnivores that eat fish. Concurrently, people unfamiliar with beavers may confuse them with otters, a species about which they may be more knowledgeable and have lived experiences, and incorrectly assume beavers consume fish.

Second, the fall of regional newspapers has coincided with the digitalisation of media, further sensationalising traditional media (Clark 2023). This is characterised by framing information as relevant, urgent, or unusual (clickbait) to compete for audience’s attention in an increasingly overcrowded digital world (Molek-Kozakowska 2013). The rise of misinformation has also been accelerated by social media and the development of echo chambers within society, limiting people’s exposure to diverse perspectives and reinforcing a shared narrative supported by a group of like-minded people (Cinelli et al. 2021). Media sensationalism may have facilitated the expansion of myths and wishful thinking about LC in different segments of society.

Third, power and institutions matter to perceptions of normality (Visoka and Lemay-Hébert 2022). Both opponents and proponents of the reintroduction of LCs have bolstered facts and beefed-up assumptions to support their view. For example, a national poll of 1071 individuals in 2020 presented participants with the statement below and asked to what extent they would support or oppose a pilot reintroduction of lynx to Scotland:

“Managing excessive deer numbers cost Forestry and Land Scotland almost £7 m in 2018/19 and almost 80 000 deer are culled annually to protect woodlands from overgrazing. Other countries in Europe, including France and Switzerland, have reintroduced lynx, a medium-sized cat which hunts deer and reduces their range: Scottish lynx were hunted to extinction at some point between 800 and 1250AD.” (Bavin and MacPherson 2022).

The poll found that 52% of people supported lynx reintroduction, and this has been used by the *Lynx to Scotland* partnership to claim that there is “widespread support” (Scotland: The Big Picture 2023, p. 2). Yet, 19% of people opposed the reintroduction and the geographic distribution of respondents is not clear. This is important because proximity to the reintroduction region is associated with less support for LC reintroductions (Ditmer et al. 2022). Proceeding without support from those who will bear the burdens of LC coexistence is likely to lead to a highly conflictual situation, such as the reintroduction of wolves in Colorado (Gonzalez et al. 2024). The statement itself also has a number of inherent problems which could sway participants towards favouring the reintroduction of lynx. First, it negatively frames deer management, informing people of the costs without stating the benefits. Second, it appears to imply that a lynx reintroduction would eliminate or significantly reduce the cost of deer management and the number of deer culled by humans. As outlined earlier, while the precise impact of a lynx reintroduction on deer populations and densities is uncertain, it is clear that in the short-term there would be a negligible impact on the cost of deer management and the number of deer culled annually. The cost of the reintroduction itself, considered in the context of deer management, may even increase the financial burden in the short-term.

Therefore, this evidence does not justify very broad claims such as “the public want lynx” (Scotland: The Big Picture 2023, p. 2). Nevertheless, the relationship between organisational and/or personal motivations, institutions, and political systems has served to legitimise and amplify messages like this one which land on more fertile ground. The combination of these three factors—the predominance of vicarious experiences, media sensationalism, and personal and political motivations for and against the reintroduction of LCs—may have driven the cultural transformation process from which the romantic and cynical discourses have emerged.

### The romantic discourse

The romantic discourse represents a vision for the future of the rural Scottish landscapes, closely associated with rewilding, which prioritises nature over human interests, and advocates for the social–ecological transformation of rural landscapes and the reintroduction of missing species (Brown et al. 2011). The principal driving force of this vision is the nature and climate crises. These crises have bred powerful feelings of loss for past landscapes and their animal and plant inhabitants (Cunsolo and Ellis 2018). However, we believe that in the absence of more reliable historical data, imaginations of past landscapes by proponents of this discourse have become nostalgic. This is characterised by the framing of past ecological conditions as pristine and people’s relationship with nature as harmonious and low impact (Monbiot 2013). The romantic discourse has weaponised nostalgic imaginations of past Scottish landscapes against rural communities to legitimise the acquisition of land, resources, and autonomy, casting contemporary land management practices as ecologically destructive (MacLeod 2023). Perhaps in response to this, Scottish rewilding organisations have sought to emphasise that people are integral to rewilding (Martin, Alison et al. 2021). Yet, there is still insufficient detail about the economic rationale for rewilding at large scales and how rewilding incorporates or benefits contemporary rural communities within these incumbent structures (Martin, Alison et al. 2021).

Lynx and wolves are treated symbolically, and their reintroduction is a vital component of the romantic discourse’s vision. Proponents of the discourse emphasise LCs’ ability to reduce and control burgeoning populations of deer, yielding vast ecological benefits by initiating trophic cascades. The reintroduction of wolves to YNP in 1995 is often cited as evidence of the wolves’ (and sometimes lynx’s) restorative powers, despite evidence disputing these claims (Brice et al. 2022; Hobbs et al. 2024; MacNulty et al. 2024). Furthermore, the romantic discourse often conceals the complexities of coexisting with LCs, idyllically portraying historical human–wolf coexistence in Britain and coexistence in Europe (Gow 2024; Why Not Scotland?, 2024). Wolves are a contentious species across Europe, with farmers facing an increased labour intensity, additional financial costs, and psychological distress (Blanco and Sundseth 2023). Trophy hunting, problem wolf hunting, and annual wolf culls occur in several countries in Europe, and lethal management is likely to increase after wolves were downgraded from “strictly protected” to “protected” under EU law (Ostermann-Miyashita et al., 2025). Ultimately, while the romantic discourse has engaged with some aspects of the social, economic, and cultural components of coexistence (e.g. tolerance, livestock protection, compensation, legal hunting), advocates have failed to work transparently and collaboratively to demonstrate how these challenges could be overcome in a Scottish context. Rural communities’ concerns about LCs have been portrayed as irrational and ill-informed. Yet, these concerns reflect the challenges and complexities of a reintroduction in a country where there is no living memory of LC coexistence.

### The cynical discourse

The cynical discourse represents an anthropocentric vision for the future of Scottish landscapes, characterised by food production and sustained support for economically and culturally significant activities such as grouse moors and deer sporting estates. This discourse emphasises the potential negative impacts of LCs’ return on recreational hunting, human safety, and livestock farming. This discourse contradicts evidence that LC range expansions do not affect deer density, especially in more highly productive environments (Melis et al. 2009; van Beeck Calkoen et al. 2023). Furthermore, in Europe and North America, where there are approximately 21 500 and 60 000 wolves, there have been 12 attacks (14 victims) of which two were fatal (both in North America) in an 18-year period between 2002 and 2020 (Linnell et al. 2021). For lynx in the same period, as far as we are aware there have been no recorded human attacks or fatalities in Europe.

Concerns about impacts on livestock farming are exemplified by the National Sheep Association’s report in response to the proposed reintroduction of lynx in Kielder Forest, which was eventually rejected in 2018. The report argues that any losses from lynx would be intolerable, proposed compensation would be insufficient, mitigation measures would not be practical, and previous lynx reintroductions in Europe have been problematic (National Sheep Association 2016). Proponents of the cynical discourse often foreground worst-case scenarios, usually Norway (e.g. Carruth 2017), where the relatively high predation rate of sheep by lynx is caused by a combination of free-ranging sheep, low populations of roe deer, and limited preventative measures (Odden et al. 2006; Blanco and Sundseth 2023). Based on these sources, many livestock producers are worried that lynx reintroduction will “eradicate farming” (Bavin and MacPherson 2022, p. 40). While on the face of it this claim, and others within the discourse, are not supported by evidence, it is vital to consider the motivation for these statements and recognise elements of truth in them. Moreover, it is important to recognise that many people making these claims may genuinely believe them, and those beliefs will influence behaviours before, during, and after any reintroduction.

The principal driving force of the cynical discourse’s characterisation of LC reintroduction might be that it is symbolic of a wider movement, strongly linked to (guerrilla) rewilding, that advocates of the cynical discourse believe will lead to less control, loss of livelihood, increased eco-tourism, and more “green lairds” (landowners associated with rewilding, carbon offsetting, and reforestation). While LCs remain largely on the periphery, other elements of this movement are being enacted and becoming more present. The growing number of rewilding projects and the covert reintroduction of animals like beavers in Scotland have been, on the whole, negatively received by actors within this discourse (Coz and Young 2020; Dolton-Thornton 2021; Martin Adrian et al. 2023; Martin, Alison et al. 2023; Holmes et al. 2024). A key reason is that these actors feel these changes have been imposed on them—something that may have been inflamed by the recent illegal releases of lynx and wild boar in the Cairngorms National Park (Whitehead 2025). Therefore, it seems that predominantly negative experiences of conservation translocations have reinforced individual and organisational actors’ attitudes within this discourse. On top of this, a combination of extreme weather, inflation, and funding cuts for nature-friendly farming has squeezed farmers financially and created greater uncertainty. Therefore, occurring within this context, any proposed LC reintroduction faces considerable challenges, especially if the process is not patient, empathetic, voluntary, and collaborative.

## Conclusion

SBS is most commonly used by conservationists to highlight loss, legitimise actions, and spark hope. Yet, it seems that as well as normalising degraded landscapes, SBS can also lead to targeting nostalgic landscapes based on anecdote, not evidence. In Scotland, these nostalgic landscapes are usually occupied by extirpated species, including LCs. This paper investigates how SBS might affect debates over extirpated species in Scotland using the potential reintroductions of lynx and wolves. We propose that both LCs have been culturally transformed in Scotland to become romanticised by some and vilified by others. The underlying motivations for actors within the romantic discourse appear to originate from powerful feelings of loss for past landscapes and extirpated species, and of the need for drastic and rapid change to combat the biodiversity and climate crises. Conversely, the underlying motivations of actors within the cynical discourse appear to originate from strong feelings of distrust towards rewilding, uncertainty about the place of traditional livelihoods in future rural landscapes, and resentment that change is being imposed on them. It is easy to look past these feelings or mischaracterise the motivations of the ‘opposing’ side because of the loudly articulated views of certain individuals and organisations which make discussions more conflictual and polarised—even though the majority of the general public may take less polarised positions (Teel and Manfredo 2010; Manfredo et al. 2021).

One reason why polarising voices are amplified and have a disproportionate influence is that claims about the social–ecological impacts of LCs and human–LC coexistence land on more fertile ground. The paucity of (scientific and historic) evidence and the loss of coexistence experience in Scottish society could mean that it is especially difficult to combat mis- and dis-information. The extinction of experience appears to be of particular importance, and so further research is needed to examine human–wildlife coexistence in places where large charismatic animals have never been extirpated to better understand this relationship. Perhaps in these locations, discourses about LCs premised on hearsay and characterised by polarisation are less prevalent because mis- and dis-information is inhibited by lived, multi-generational experiences of coexistence. This may provide new evidence about human–wildlife coexistence that could inform management of naturally recolonising populations and reintroductions of extirpated species in Scotland and beyond.

However, facts alone do not change minds. Even the availability of very good data that supports or undermines any LC reintroduction is unlikely to convince people since the debate is often not about LCs, but about what they represent—a deep-rooted conflict over how future rural Scottish landscapes should look, what purpose they should serve, and who should control them. Therefore, the main focus of the conservation community must be on enacting long-term processes of facilitated dialogue that look beyond ecological baselines and engage with the multiple social, cultural, and other baselines entangled with ecological change.

For example, any LC translocation must not only grapple with the ecological challenges of reintroduction but consider the social and cultural features of landscapes that are critical for fostering favourable coexistence. Looking beyond ecological baselines in this context necessitates a more inclusive and collaborative approach to successfully introduce new social and cultural features of landscapes by actively working with farmers and other rural interests in Scotland. Furthermore, meaningfully engaging with social and cultural baselines may require the immediate focus of the rewilding community to shift from LCs to embedding social justice principles into practice to ensure that land-use transitions are locally beneficial, sensitive, and participatory (Martin, Adrian et al. 2023, Martin, Alison et al. 2023). This aligns with calls for rewilding to be pragmatic and work iteratively, whereby “appropriate interventions are applied successively to progress a system towards a bold vision”, i.e. the reintroduction of LCs (Hawkins et al. 2024, p. 4). This process should be viewed as an opportunity to carefully co-produce visions of rural landscapes, with or without LCs, that retain ecological ambition, respect differing values, and support multiple land uses.

## Data Availability

No data were used for the research described in the article.
