# Correction: A shifting baseline theory of debates over potential lynx and wolf reintroductions to Scotland

**DOI:** 10.1007/s13280-025-02257-y

**Published:** 2025-09-27

**Authors:** Toryn Whitehead, Darragh Hare

**Affiliations:** 1 Department of Geography, King’s College London, 40 Aldwych, London, WC2B 4BG UK; 2 Wildlife Conservation Research Unit, Department of Biology, The Recanati-Kaplan Centre, University of Oxford, Oxford, UK; 3 Leverhulme Centre for Nature Recovery, School of Geography and the Environment, University of Oxford, Oxford, UK; 4 Department of Natural Resources and the Environment, Cornell University, Ithaca, NY USA

**Correction to: Ambio 2025, 54: 1598–1610** 10.1007/s13280-025-02186-w

In the original article, in the third paragraph of the **Introduction**, the authors incorrectly stated that the Missing Lynx Project and the Lynx to Scotland project currently propose a five-year trial reintroduction of lynx. The sentences “At present, *The Missing Lynx Project* proposes a five-year trial reintroduction of lynx in Northumbria, England, to forest land that extends into the Scottish Borders. The Lynx to Scotland project, comprising three rewilding organisations, also proposes a five-year trial reintroduction of lynx in the Scottish Highlands” should have been “At present, *The Missing Lynx Project* is exploring the feasibility of a lynx reintroduction in Northumberland, England, into a habitat patch which also includes bordering areas of southern Scotland. The Lynx to Scotland project, comprising three rewilding organisations, proposes a phased release programme with annual check points, over at least a five-year period, in the Scottish Highlands”.

And Darragh Hare’s author biography statement should be corrected from “Darragh Hare is a research fellow in the Wildlife Conservation Research Unit (WildCRU) in the Zoology Department at Oxford University. His research interests include morality and biodiversity conservation” to “Darragh Hare is a research fellow in the Wildlife Conservation Research Unit (WildCRU) in the Biology Department at Oxford University. His research interests include social conflicts over biodiversity conservation”.

